# Alignment between the patient’s cancer worry and the GP’s cancer suspicion and the association with the interval between first symptom presentation and referral: a cross-sectional study in Denmark

**DOI:** 10.1186/s12875-021-01480-2

**Published:** 2021-06-24

**Authors:** Line Flytkjær Virgilsen, Anette Fischer Pedersen, Peter Vedsted, Gitte Stentebjerg Petersen, Henry Jensen

**Affiliations:** 1 Research Centre for Cancer Diagnosis in Primary Care, Research Unit for General Practice, Bartholins Allé 2, 8000 Aarhus C, Denmark; 2 Department of Clinical Medicine, Aarhus University, Palle Juul-Jensens Boulevard 82, 8200 Aarhus C, Denmark; 3 Danish Cancer Society, Strandboulevarden 49,, 2100 København Ø, Denmark

**Keywords:** Neoplasms, Denmark, General practice, Early diagnosis, Primary health care, Signs and symptoms

## Abstract

**Background:**

General practitioners (GPs) have a key role in the diagnosis of cancer. It is crucial to identify factors influencing the decision to refer for suspected cancer. The aim of this study was to investigate the alignment between the patient’s cancer worry and the GP’s suspicion of cancer in the first clinical encounter and the association with the time interval from the first symptom presentation until the first referral to specialist care, i.e. the primary care interval (PCI).

**Method:**

The study was performed as a cross-sectional study using survey data on patients diagnosed with incident cancer in 2010 or 2016 and their GPs in Denmark. We defined four alignment groups: 1) patient worry and GP suspicion, 2)  GP suspicion only, 3) patient worry only, and 4) none of the two. A long PCI was defined as an interval longer than the 75th percentile.

**Results:**

Among the 3333 included patients, both patient worry and GP suspicion was seen in 39.5%, only GP suspicion was seen in 28.2%, only patient worry was seen in 13.6%, and neither patient worry nor GP suspicion was seen in 18.2%. The highest likelihood of long PCI was observed in group 4 (group 4 vs. group 1: PPR 3.99 (95% CI 3.34–4.75)), mostly pronounced for easy-to-diagnose cancer types.

**Conclusion:**

Misalignment between the patient’s worry and the GP’s suspicion was common at the first cancer-related encounter. Importance should be given to the patient interview, due to a potential delayed GP referral among patients diagnosed with “easy-to-diagnose” cancer types presenting with unspecific symptoms.

**Supplementary Information:**

The online version contains supplementary material available at 10.1186/s12875-021-01480-2.

## Introduction

Cancer incidence is increasing in most western countries [1, 2]. At the same time, improvements in the prognosis are seen in patients with cancer, among others because of better treatment options [3] and increased focus on cancer diagnostic strategies, including the implementation of cancer patient pathways (CPPs) [4–6].

In healthcare systems relying on the GP to act as gatekeeper, the majority of patients with cancer initially present their symptoms and signs in general practice [7–11]. In these countries, the GP plays a central role in ensuring early diagnosis of cancer [4]. The time from the patient’s symptom presentation in general practice until the referral by the GP for further specialised diagnostic investigations, i.e. the primary care interval (PCI), is often short, with median intervals ranging from 0 to 20 days across cancer types (e.g. 14 days for lung cancer and 0 days for breast cancer) [12]. Research has investigated patient and GP factors of importance for prompt GP referral [13, 14], yet, more knowledge is needed on the triggers of GP referral [7].

The positive predictive value of cancer is low for most symptoms, including alarm symptoms [15]. Thus, the factors leading to GP suspicion can be crucial in the diagnostic pathway. Research has shown that the GP’s suspicion depends among others on age, symptom presentation and GP experience [8, 16]. Our research group has found that the patient’s own worry about cancer when presenting symptoms is associated with the GP’s suspicion of cancer [17]. Moreover, the GP’s suspicion of cancer has been identified as an important factor for referral to a CPP and for experiencing a shorter diagnostic interval [18]. A qualitative study from the United Kingdom found that the diagnostic timeliness in general practice could be affected by the degree of alignment between the patient’s and the GP’s symptom perception [19]. Yet, little is known about how the patient’s worry interacts with the GP’s cancer suspicion in the first clinical encounter, and how this interaction is associated with the time to referral from general practice.

This study aimed to investigate if the alignment between the patient’s cancer worry and the GP’s cancer suspicion at the first clinical presentation of symptoms was associated with a long PCI. We hypothesised that non-alignment was associated with a prolonged PCI and that the PCI was most prolonged in the absence of patient worry and GP suspicion.

## Method

### Setting

Denmark has a population of 5.8 million people. The healthcare system is tax-funded and based on the principle of free and equal access for all citizens. GPs act as gatekeepers to specialised healthcare, and 98% of Danish citizens are registered with a general practice. In the two recent decades, several initiatives have transformed the diagnosis and treatment of cancer in Denmark, e.g. the introduction of CPPs [6].

### Population and data collection

This population-based cross-sectional study was based on a population of patients (aged ≥18 years) recorded in the Danish National Patient Register (DNPR) [20] with a diagnosis of first-time cancer, excluding non-malignant melanoma (ICD-10: C43), in the spring of 2010 or autumn of 2016 and listed with a GP in Denmark (*n* = 19,996). Data included questionnaire information from both patients and their GPs.

Approximately 3–6 months after the diagnosis, alive patients received a questionnaire about the time from first symptom until start of treatment if available for research studies according to the Danish Civil Registration System [21] (*n* = 16,591) (patient response rate: 56.7%).

The registered GP of each patient was asked to complete a questionnaire after identification through the Danish National Health Service Register [22] (GP response rate: 74.1%). In 2010, the GPs of all identified patients received a questionnaire, following permission from the Danish National Board of Health in accordance with the Danish Health Act. Questionnaires were sent to GPs at 2–5 weeks after patient identification [23]. In 2016, patient consent was required by law before contacting their GP. Thus, the GP received the questionnaire after patient consent at 3–12 months after patient identification.

**Fig. 1:**
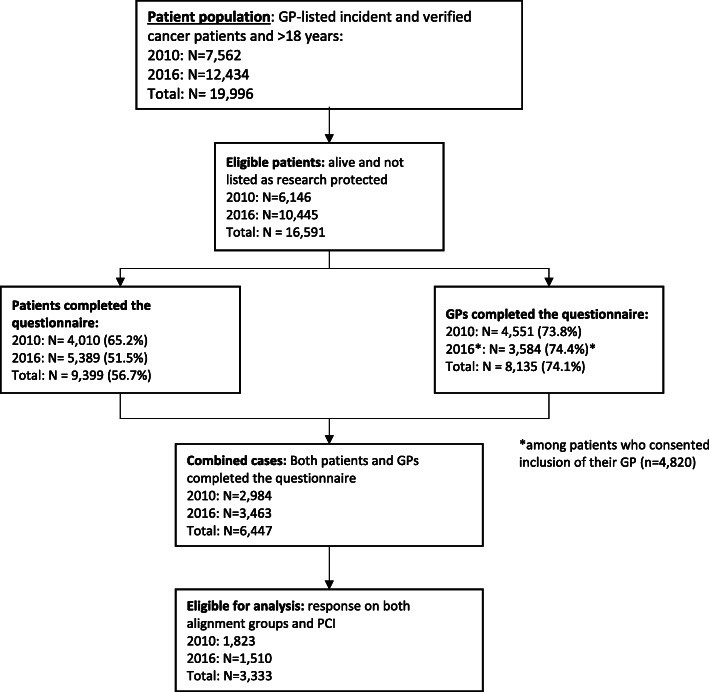
Flowchart of the study population

In this study, inclusion required three criteria to have been fulfilled. First, the patient responded to the “cancer worry” item. Second, the patient’s GP responded to the “suspicion” item. Third, valid data was available on the primary care interval (*n* = 3333) (Fig. 1).

### Main variables

All data used in this study was linked through the unique civil registration (CRN) number [21].

#### Alignment between patient’s cancer worry and GP’s cancer suspicion

Groups of alignment were defined according to the agreement between the patient’s cancer worry and the GP’s suspicion of cancer or a serious illness. Patients were asked” Were you worried that you might have cancer when consulting your GP for the first time?” The response options “A little”, “A great deal” and “Very much” were combined into “Worried” and compared with the response option “No”. The GPs were asked: “When the patient consulted general practice for the first time, what was your overall evaluation of the patient?” The response options “I/my practice suspected cancer” and “I/my practice suspected serious illness, but not specifically cancer” were combined and compared to the response option “I/my practice did not directly suspect cancer or a serious illness”. Four alignment groups were formed on the basis of the patient’s cancer worry and the GP’s suspicion of cancer or serious illness at the first clinical encounter.

#### Primary care interval

The primary care interval (PCI) was defined as the number of days between first symptom presentation until first referral to specialist care as outlined in the Aarhus Statement [24]. Data was obtained from the GP questionnaire and based on two questions “On which date did the patient consult you/your clinic for the first time due to discomfort/symptoms, which ─ based on your current knowledge ─ is likely to have been caused by the patient’s cancer disease?” and “On which date did you/your clinic refer the patient for the first time to further investigation at a private practice specialist or hospital, thereby transferring the responsibility for the patient’s diagnostic pathway to another healthcare unit?” When data was missing from the GP, the corresponding date reported by the patient was used. PCIs with negative values were coded as 0 days, and PCIs longer than 365 days were coded as 365 days in accordance with previous research [25].

#### Covariates

From the Danish Civil Registration System, we obtained information on the patient’s sex and age. From Statistics Denmark, we obtained information on the patient’s education and marital status. Marital status was dichotomised into married and unmarried. The patient’s highest attained education in the year of diagnosis was defined according to the International Standard Classification of Education (ISCED) [26], and divided into short (≤10 years), medium (11–15 years) and long education (> 15 years). Charlson’s Comorbidity Index (CCI) following the method by Quan et al. [27] was used and calculated from diagnosis registrations in the National Patient Registry for up to 10 years before the cancer diagnosis and categorised into no (CCI: 0), low (CCI: 1–2) and high (CCI: > 2). Diagnostic difficulty was categorised into easy, intermediate and hard to diagnose cancer types [13, 28–30] (Additional file 1).

#### Statistical analysis

Prior to study initiation, a power calculation was performed to compare the reference group (group 1, n: 1316) with the smallest group (group 3, n: 452). Assuming that 25% of patients in group 1 have a long interval [31], we needed α = 0.05 and power = 1.0 to be able to detect a difference of 30 percentage points in the proportion of having a long interval between group 1 and group 3.

As no legally decided or scientifically accepted PCI is applied across cancer sites in Denmark, the data distribution was used to define a long PCI. Thus, a long PCI was defined as a PCI longer than the 75th percentile, corresponding to 15 days. We analysed the association between the alignment of patient’s cancer worry and GP’s suspicion of cancer or serious illness and long PCI, using generalised linear models (GLM) with log link for the Poisson family with the outcome presented as prevalence rate ratios (PRR) [32, 33] with 95% confidence intervals (95% CI). Robust variance estimates according to GP provider number were used to allow for clustering of patients by general practice in both unadjusted and adjusted models. In all analyses, group 1 (worried patients and suspicious GPs) served as the reference group.

**Fig. 2:**
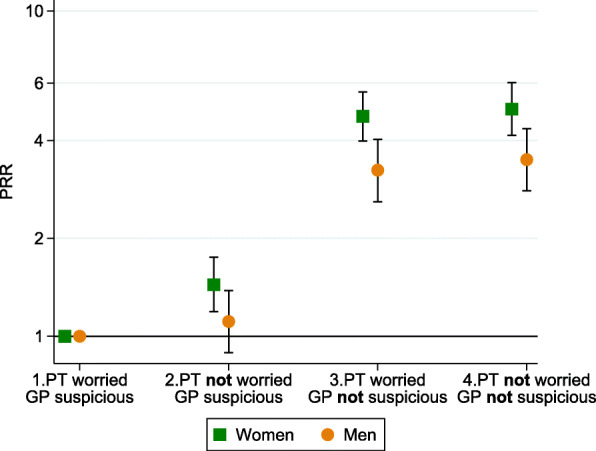
Association between the alignment between patient worry and GP suspicion and the patient having a long PCI expressed as prevalence rate ratios (PRRs); stratified by sex (*n* = 3333). Reference group= “1. Patient worried about cancer and the GP suspected cancer”. Long PCI for each strata is defined based on a cut-off at the 75th percentile for each group, i.e. 21 days for males and 8 days for women. Adjusted for sex, age, year of diagnosis, education, marital status, CCI

**Fig. 3:**
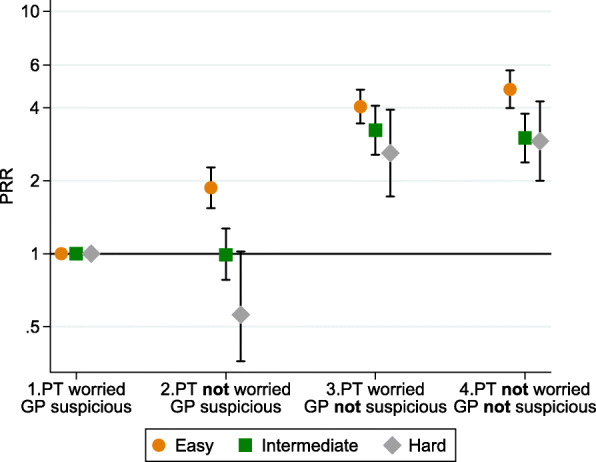
Association between the alignment between patient worry and GP suspicion and the patient having a long PCI expressed as prevalence rate ratios (PRRs); stratified by diagnostic difficulty (*n* = 3333). Reference group: Patient worried about cancer and GP suspected cancer (group 1). Long PCI for strata is defined based on cut-off at the 75th percentile for each group, i.e. 3 days for easy, 26 days for intermediate and 30 days for hard-to-diagnose cancer. Adjusted for sex, age, year of diagnosis, education, marital status, CCI

Further, adjusted GLM models with interaction terms were used to test the interaction between the covariates. Sex and diagnostic difficulty interacted in the model, and GLM models for the association between alignment groups and PCI were run stratified for each strata in these variables. The results are presented graphically (Figs. 2 and 3). As the PCI differed within these groups, long PCI was based on the cut-off value at the 75th percentile for each group, e.g. the 75th percentile was 8 days for females and 21 days for males.

Two sub-analyses were conducted (Additional files 2 and 3). First, we investigated the alignment between patient’s cancer worry and GP’s suspicion and the association with long PCI when excluding gender-specific cancers (breast, gynaecological, prostate and testis cancers). Second, we stratified the analyses on cancer type. All analyses were performed in Stata 15.

## Results

Table 1 shows the formation and distribution of the four alignment groups. The highest proportion of the population was between 60 and 69 years, married, had medium to long education, diagnosed with an easy-to-diagnose cancer type and had no comorbidity (Table 2). The median PCI was 0 days (Interquartile interval (IQI): 0 (0) 15).

**Table 1:** The formation of alignment groups according to patient’s cancer worry at first GP presentation and GP’s suspicion of cancer or serious illness at the first consultation (*n* = 3333)

	GP suspicious		GP not suspicious		Total	
	N	% (column)	N	% (column)	N	% (column)
**The patient was**						
Worried	***Group 1***		***Group3***			
	1316	(57.8)	452	(41.3)	1768	(52.2)
Not worried	***Group 2***		***Group 4***			
	960	(42.2)	605	(58.7)	1565	(46.9)
Total	2276	(100.0)	1057	(100.0)	3333	(100)

**Table 2:** Distribution of alignment groups and patient characteristics (*n* = 3333)

	Total^a^	
	n	(%)
**Total**	3333	(100)
**Patient (PT)-GP alignment**		
1.PT worried, GP suspicious	1316	(39.5)
2.PT *not* worried, GP suspicious	960	(28.2)
3.PT worried, GP *not* suspicious	452	(13.6)
4.PT *not* worried, GP *not* suspicious	605	(18.2)
**Age group (years)**		
18–49	438	(13.1)
50–59	545	(16.4)
60–69	1027	(30.8)
70–79	969	(29.1)
> 80	354	(10.6)
**Sex**		
Female	1654	(49.6)
Male	1679	(50.4)
**Year of diagnosis**		
2010	1823	(54.7)
2016	1510	(45.3)
**Marital status**		
Cohabiting/married	2252	(67.6)
Living alone	1079	(32.4)
**Education**		
Short (≤10 years)	986	(30.1)
Medium (11–15 years)	1382	(42.2)
Long (> 15 years)	904	(27.6)
**Diagnostic difficulty of cancer**^**b**^		
Easy	1502	(47.2)
Intermediate	1155	(36.3)
Hard	522	(16.4)
**Comorbidity (CCI**^**c**^**score)**		
None (0)	2312	(74.1)
Low (1–2)	673	(21.6)
High (2 or more)	134	(4.3)
**Referral to cancer patient pathway**		
Yes	1809	(43.5)
No	2348	(56.5)

^a^Numbers vary due to missing data

^b^ See previous research [13, 28–30]

^c^ Charlson Comorbidity Index

In 39.5% of the 3333 included patients, the patient had worried about and the GP had suspected cancer or a serious illness at the first consultation (group 1). In 18.2% of patients, the patient had not worried about cancer and the GP had not suspected cancer or a serious illness at the first clinical presentation (group 4). Misalignment occurred in four out of 10 patients (Table 2). Further, a higher proportion of patients in group 4 had hard-to-diagnose cancer types and were in the age groups 50–70 years, but no other significant differences were seen between patient characteristics and alignment groups based on Pearson’s chi square test (data not shown).

Patient-GP alignment was strongly associated with PCI (Table 3). When neither the patient worried about cancer nor the GP suspected cancer or a serious illness (group 4), patients were four times more likely to have a long PCI (PRR 3.99 (95% CI 3.34–4.75)) than when both the patient worried and the GP suspected cancer or a serious illness. The likelihood of having a long PCI was almost similar for group 3, where the patient worried and the GP did *not* suspect cancer or a serious illness. Likewise, in group 2, where the patient did *not* worry, but the GP was suspicious, increased likelihood of having a long PCI was seen (PRR 1.30 (95% CI 1.12–1.50)) (Table 3).

**Table 3:** Prevalence rate ratio (PRR) of having long PCI according to the patient-GP alignment in the first clinical encounter and patient characteristics (*n* = 3333)

			PRR of having long PCI			
	Long PCI		Unadjusted		Adjusted^**a**^	
	N	(%)	PRR	95% CI	PRR	95% CI
**Patient (PT)-GP alignment**						
1.PT worried, GP suspicious	142	(10.8)	1		1	
2.PT *not* worried, GP suspicious	159	(16.6)	**1.53**	**(1.34–1.76)**	**1.30**	**(1.12–1.50)**
3.PT worried, GP *not* suspicious	218	(48.2)	**4.47**	**(3.86–5.18)**	**3.70**	**(3.21–4.26)**
4.PT *not* worried, GP *not* suspicious	321	(53.1)	**4.92**	**(4.16–5.82)**	**3.99**	**(3.34–4.75)**
**Sex**						
Male	513	(31.0)	**1**		**1**	
Female	327	(19.5)	**0.65**	**(0.57–0.73)**	**0.81**	**(0.70–0.92)**
**Age groups (years)**						
18–49	67	(15.3)	1		**1**	
50–59	127	(23.3)	**1.52**	**(1.19–1.95)**	1.13	(0.84–1.53)
60–69	269	(26.2)	**1.71**	**(1.41–2.08)**	1.28	(0.94–1.73)
70–79	282	(29.2)	**1.90**	**(1.56–2.31)**	**1.36**	**(1.02–1.81)**
> 80	95	(26.8)	**1.75**	**(1.40–2.21)**	**1.45**	**(1.19–1.76)**
**Year of diagnosis**						
2010	476	(26.1)	1		1	
2016	364	(24.1)	0.92	(0.92–1.04)	**0.89**	**(0.82–0.96)**
**Marital status**						
Cohabiting/Married	577	(25.6)	1		1	
Not married	263	(24.4)	0.95	(0.86–1.05)	1.03	(0.96–1.13)
**Education**						
Short (≤10 years)	284	(28.8)	**1**		1	
Medium (11–15 years)	329	(23.8)	**0.83**	**(0.75–0.91)**	**0.85**	**(0.78–0.93)**
Long (> 15 years)	218	(24.1)	**0.84**	**(0.75–0.93)**	0.92	(0.81–1.02)
**Diagnostic difficulty**^**b**^						
Easy	230	(15.3)	1		1	
Intermediate	383	(33.2)	**2.17**	**(1.92–2.44)**	**1.53**	**(1.37–1.70)**
Hard	189	(36.2)	**2.36**	**(2.09–2.68)**	**1.67**	**(1.48–1.87)**
**Comorbidity (CCI**^**c**^ **score)**						
None (0)	563	(24.4)	1		1	
Low (1–2)	183	(27.2)	1.12	(0.93–1.34)	0.95	(0.81–1.12)
High (2 or more)	41	(30.6)	**1.26**	**(1.04–1.51)**	1.01	(0.84–1.22)

Significant results are shown in bold

^a^Adjusted for sex, age, year of diagnosis, marital status, education, diagnostic difficulty and CCI

^b^ See previous research [13, 28–30]

^c^ Charlson Comorbidity Index

When stratifying on sex, we found that non-worried females with a non-suspicious GP (group 4) had high probability of having a long PCI (PRR 4.99 (95% CI 4.14–6.02)) (Fig. 2). Easy-to-diagnose cancer types had the shortest PCI (25% had longer PCI than 3 days), and hard-to-diagnose cancer types had the longest PCI (25% had longer PCI than 30 days). When the patient did not worry about cancer and the GP did not suspect cancer or a serious illness at the first presentation, the PCI was particularly elevated among cancer patients with easy-to-diagnose cancer types (PRR 4.76 (95% CI 3.98–5.70))(Fig. 3).

### Sensitivity analysis

When we excluded the gender-specific cancer types, the estimates were reduced, but they remained statistically significant for group 3 and group 4, but not for group 2 (Additional file 2). Stratified analyses on cancer types showed that the PCI was elevated in group 4, specifically in patients with breast cancer (Additional file 3).

## Discussion

### Summary

This study showed that misalignment between patient worry and GP suspicion occurred at the first clinical presentation in four of ten cancer patients. For almost one in five cancer patients, neither the patient nor GP reported concerns about cancer. For 14% of the cancer patients, only the patient was concerned, and the prolonged PCI in this group was comparable to that in the group with neither patient worry nor GP suspicion, which was most pronounced among easy-to-diagnose cancer types. Misalignment increased the likelihood of having a prolonged PCI. However, absence of GP suspicion seemed to impact the PCI more than absence of worry in the patient.

### Strengths and limitations

This population-based study included all first-time cancer patients diagnosed in the spring of 2010 or autumn of 2016, and no patients were excluded a priori. Some of the identified alive patients (*n* = 16,591) were not possible to include in the analysis, thus, selection bias is plausible. Still, not all identified patients were in the target group for this study as a proportion (about 25% [8]) bypassed the GP in the diagnostic route. Selection bias could further have occurred as some patients died before receiving the questionnaire, and this group may have differed in terms of patient worry, GP suspicion and PCI. Moreover, non-respondents in surveys are known to have low socioeconomic status, which could influence prevalence measures but often does not markedly affect estimates [34].

Recall bias is common in retrospective questionnaire data [35, 36]. This could have occurred in the 2016 cohort as some GPs received the questionnaire for up to 1 year after the diagnosis. However, the information on the PCI relied mainly on the GP’s electronic medical records. Therefore, recall bias is most likely to have been present if patients presented with unspecific symptoms. The risk of misclassifying the PCI was reduced by replacing missing dates in the GP questionnaire with patient-assessed dates [37]. Data on cancer worry among patients was assessed after the diagnosis of cancer had been established. Some patients might have had difficulties remembering the exact time in the diagnostic process when they became worried.

This study had no access to systematically collected information on the patient’s symptom presentation, which could have strengthened the insights of the findings and could have made it possible to distinguish between presented symptoms and the GP’s level of suspicion. Yet, although symptom presentation is crucial for the GP’s assessment in the clinical encounter [16], research has emphasised that the suspicion in the GP is complex and depends on several factors, such as patient age, comorbidity, previous GP attendance and gut feeling [16, 18, 38]. The project also had no information on current health campaigns at the time of the data collection, which could have altered the patient’s worry perception. Yet, as most of the existing CPPs had been implemented in Denmark by 2010, we do not expect that changes in the healthcare system over time could affect the results. This was supported in our results as no significant difference was seen between alignment groups and PCI in the two data collection periods, and the PCI was also stable over time (IQI in 2010: 0 (0) 15 days, IQI in 2016: 0 (0) 14 days).

### Comparison with existing literature

To our knowledge, no previous studies have quantified how the alignment between the patient’s cancer worry and the GP’s cancer suspicion is associated with the PCI. The results showed that the PCI was most prolonged when the patient did not worry about cancer and the GP did not suspect cancer or a serious illness (group 4), e.g. 25% in group 1 had a PCI of 2 days or longer, whereas 25% in group 4 had a PCI of 60 days or longer. A qualitative study found that misalignment is common between patients and GPs in the perception of a presented symptom, and the authors also found that this misalignment might affect the timeliness of the cancer diagnosis [19]. This is in line with our finding in the present study that four in ten patients were categorised in a group with misalignment and spent longer time in primary care before referral. As the strongest association was observed in patient groups with absence of GP suspicion (groups 3 and 4), the results are consistent with the literature on the GP’s gut feeling; a recent meta-analysis reported a four times increased cancer risk if the GP had reported a gut feeling of cancer [39]. Thus, this underlines that the GP’s suspicion is crucial for the diagnostic strategy, even if it does not align with the patient’s perception.

Although multiple factors affect whether a symptom is perceived as serious or worrisome by the patient [40, 41], some research has indicated that alarm symptoms are more often than non-alarm symptoms perceived as something potentially serious [42]. Additionally, the GP is more likely to suspect cancer or a serious illness when alarm symptoms are presented [16, 38]. However, not all patients present with symptoms qualifying for an urgent referral [43, 44], which in turn may increase the waiting time in primary care [45]. Presentation of atypical symptoms may make the GP susceptible to cognitive bias [46], which can be triggered by reliance on “rules-of-thump” practices that may be defective. These cognitive mechanisms may be reinforced when dealing with easy-to-diagnose cancers where the GP may have especially clear expectations to how the cancer disease *should* present. This hypothesis could explain the almost fivefold increased likelihood of long PCI for easy-to-diagnose cancer types when the first consultation was characterised by absence of both patient worry and GP suspicion. It could also explain previous findings that some patients with easy-to-diagnose cancers had more than three consultations in general practice before diagnosis [31].

The link between alignment of patient worry and GP suspicion and the PCI was strongest among female cancer patients. This could be related to the strong association between absence of patient worry and GP suspicion (group 4) and prolonged PCI among breast cancer patients, at least to some degree. Some studies have indicated that patients presenting with symptoms other than “lump” tend to experience delays in healthcare seeking [47] and prolonged PCI [47, 48] in the diagnosis of breast cancer.

### Implications

In 28%, the patient did not worry, but the GP did suspect cancer or a serious illness (group 2). A prolonged PCI was seen in this group, which could indicate that the absence of patient worry somehow convinced the GP to adopt a wait-and-see or similar strategy that prolonged the PCI. This finding clearly stresses the need for safety netting when the GP uses a wait-and-see strategy despite vague suspicion of serious disease. Yet, the results suggest that the PCI is mostly prolonged when the GP’s suspicion is absent. A report among Danish cancer patients found that patients do not always express their worry when they present potential signs of cancer to their GP [49]. This emphasises the significance of the patient interview in the clinical encounter as patient worry was present in 14%, yet suspicion was absent in the GP (i.e. group 3).

The low level of suspicion seen for easy-to-diagnose cancers with atypical presentation is of clinical interest. It indicates a need for increased focus on GP training, use of safety netting and higher awareness of symptom significance, especially in patients with multiple consultations in primary care before referral [13], in order to lower the risk of prolonged diagnostic time intervals in primary care [50].

## Conclusion

In six of ten cancer patients presenting in general practice, cancer or another serious disease was not considered by the patient, the GP or both at the first presentation. Misalignment was common and associated with prolonged PCI. Although GP suspicion remains crucial for the time to referral, this study underlines the importance of the patient interview in the diagnosis of cancer in primary care. The highest likelihood of prolonged PCI was observed when the patient did not worry about cancer and the GP did not suspect cancer or a serious illness at the first clinical presentation. Finally, patients presenting with an easy-to-diagnose type of cancer had higher likelihood of experiencing a long PCI in cases characterised by no patient worry and no GP suspicion.

## Supplementary Information

**Additional file 1 **: **Table 1**: Categorisation of cancer types according to how difficult they are to diagnose clinically, based upon number of contacts in primary care before diagnosis in England [13, 28–30].

**Additional file 2 **Prevalence rate ratio (PRR) of having long PCI according to the alignment between patient (PT) and general practitioner (GP) at the first clinical encounter and patient characteristics excluding gender-specific cancer types (*n* = 1639).

**Additional file 3 **Adjusted* prevalence rate ratio (PRR) of having long PCI according to the alignment between patient (PT) and general practitioner (GP) in the first clinical encounter and patient characteristics stratified on cancer type (*n* = 3333).

## Data Availability

The datasets generated and analysed in the current study are not publicly available as the data is stored at Statistic Denmark and is only accessible through a protected network connection in accordance with the Danish regulations of research.

## Abbreviations

CCI: Charlson’s Comorbidity Index
CI: Confidence interval
CPP: Cancer patient pathway
CRN: Civil registration number
DNPR: Danish National Patient Register
GLM: Generalised linear models
GP: General practitioner
ICD-10: International Classification of Diseases
ISCED: International Standard Classification of Education
IQI: Interquartile interval
PCI: Primary care interval
PRR: Prevalence rate ratio

## References

[1] Torre LA, Siegel RL, Ward EM, Jemal A (2016). Global Cancer incidence and mortality rates and trends--an update. Cancer Epidemiol Biomark Prev.

[2] Allemani C, Weir HK, Carreira H, Harewood R, Spika D, Wang XS, Bannon F, Ahn JV, Johnson CJ, Bonaventure A, Marcos-Gragera R, Stiller C, Azevedo e Silva G, Chen WQ, Ogunbiyi OJ, Rachet B, Soeberg MJ, You H, Matsuda T, Bielska-Lasota M, Storm H, Tucker TC, Coleman MP, CONCORD Working Group (2015). Global surveillance of cancer survival 1995-2009: analysis of individual data for 25,676,887 patients from 279 population-based registries in 67 countries (CONCORD-2). Lancet.

[3] Arnold M, Rutherford MJ, Bardot A, Ferlay J, Andersson TM, Myklebust TA, Tervonen H, Thursfield V, Ransom D, Shack L (2019). Progress in cancer survival, mortality, and incidence in seven high-income countries 1995-2014 (ICBP SURVMARK-2): a population-based study. Lancet Oncol.

[4] Emery JD, Shaw K, Williams B, Mazza D, Fallon-Ferguson J, Varlow M, Trevena LJ (2014). The role of primary care in early detection and follow-up of cancer. Nat Rev Clin Oncol.

[5] Olesen F, Hansen RP, Vedsted P (2009). Delay in diagnosis: the experience in Denmark. Br J Cancer.

[6] Probst HB, Hussain ZB, Andersen O (2012). Cancer patient pathways in Denmark as a joint effort between bureaucrats, health professionals and politicians--a national Danish project. Health Policy.

[7] Rubin G, Berendsen A, Crawford SM, Dommett R, Earle C, Emery J, Fahey T, Grassi L, Grunfeld E, Gupta S, Hamilton W, Hiom S, Hunter D, Lyratzopoulos G, Macleod U, Mason R, Mitchell G, Neal RD, Peake M, Roland M, Seifert B, Sisler J, Sussman J, Taplin S, Vedsted P, Voruganti T, Walter F, Wardle J, Watson E, Weller D, Wender R, Whelan J, Whitlock J, Wilkinson C, de Wit N, Zimmermann C (2015). The expanding role of primary care in cancer control. Lancet Oncol.

[8] Jensen H, Torring ML, Olesen F, Overgaard J, Vedsted P (2014). Cancer suspicion in general practice, urgent referral and time to diagnosis: a population-based GP survey and registry study. BMC Cancer.

[9] Weller D, Menon U, Zalounina Falborg A, Jensen H, Barisic A, Knudsen AK, Bergin RJ, Brewster DH, Cairnduff V, Gavin AT, Grunfeld E, Harland E, Lambe M, Law RJ, Lin Y, Malmberg M, Turner D, Neal RD, White V, Harrison S, Reguilon I, Vedsted P, ICBP Module 4 Working Group (2018). Diagnostic routes and time intervals for patients with colorectal cancer in 10 international jurisdictions; findings from a cross-sectional study from the international Cancer benchmarking partnership (ICBP). BMJ Open.

[10] Elliss-Brookes L, McPhail S, Ives A, Greenslade M, Shelton J, Hiom S, Richards M (2012). Routes to diagnosis for cancer - determining the patient journey using multiple routine data sets. Br J Cancer.

[11] Allgar VL, Neal RD (2005). General practictioners’ management of cancer in England: secondary analysis of data from the National Survey of NHS patients-Cancer. Eur J Cancer Care.

[12] Lyratzopoulos G, Saunders CL, Abel GA, McPhail S, Neal RD, Wardle J, Rubin GP (2015). The relative length of the patient and the primary care interval in patients with 28 common and rarer cancers. Br J Cancer.

[13] Lyratzopoulos G, Neal RD, Barbiere JM, Rubin GP, Abel GA (2012). Variation in number of general practitioner consultations before hospital referral for cancer: findings from the 2010 National Cancer Patient Experience Survey in England. Lancet Oncol.

[14] Hansen RP, Vedsted P, Sokolowski I, Sondergaard J, Olesen F (2011). General practitioner characteristics and delay in cancer diagnosis. a population-based cohort study. BMC Fam Pract.

[15] Hamilton W (2009). The CAPER studies: five case-control studies aimed at identifying and quantifying the risk of cancer in symptomatic primary care patients. Br J Cancer.

[16] Scheel BI, Ingebrigtsen SG, Thorsen T, Holtedahl K (2013). Cancer suspicion in general practice: the role of symptoms and patient characteristics, and their association with subsequent cancer. Br J Gen Pract.

[17] Virgilsen LF, Jensen H, Pedersen AF, Zalounina Falborg A, Vedsted P. Patient's worry about cancer and the general practitioner's suspicion of cancer or serious illness: A population-based study in Denmark. Eur J Cancer Care. 2021;30(3):e13411. 10.1111/ecc.13411. 10.1111/ecc.13411 33511723

[18] Jensen H, Merrild CH, Moller H, Vedsted P (2019). Association between GPs’ suspicion of cancer and patients’ usual consultation pattern in primary care: a cross-sectional study. Br J Gen Pract.

[19] Amelung D, Whitaker KL, Lennard D, Ogden M, Sheringham J, Zhou Y, Walter FM, Singh H, Vincent C, Black G (2020). Influence of doctor-patient conversations on behaviours of patients presenting to primary care with new or persistent symptoms: a video observation study. BMJ Qual Saf.

[20] Lynge E, Sandegaard JL, Rebolj M (2011). The Danish National Patient Register. Scand J Public Health.

[21] Pedersen CB (2011). The Danish civil registration system. Scand J Public Health.

[22] Andersen JS, Olivarius F, Krasnik A (2011). The Danish National Health Service Register. Scand J Public Health.

[23] Jensen H, Torring ML, Larsen MB, Vedsted P (2014). Existing data sources for clinical epidemiology: Danish cancer in primary care cohort. Clin Epidemiol.

[24] Weller D, Vedsted P, Rubin G, Walter FM, Emery J, Scott S, Campbell C, Andersen RS, Hamilton W, Olesen F, Rose P, Nafees S, van Rijswijk E, Hiom S, Muth C, Beyer M, Neal RD (2012). The Aarhus statement: improving design and reporting of studies on early cancer diagnosis. Br J Cancer.

[25] Weller D, Vedsted P, Anandan C, Zalounina A, Fourkala EO, Desai R, Liston W, Jensen H, Barisic A, Gavin A, Grunfeld E, Lambe M, Law RJ, Malmberg M, Neal RD, Kalsi J, Turner D, White V, Bomb M, Menon U, ICBP Module 4 Working Group* (2016). An investigation of routes to cancer diagnosis in 10 international jurisdictions, as part of The International Cancer Benchmarking Partnership: survey development and implementation. BMJ Open.

[26] UNESCO (2014). ISCED: International Standard Classification of Education.

[27] Quan H, Li B, Couris CM, Fushimi K, Graham P, Hider P, Januel JM, Sundararajan V (2011). Updating and validating the Charlson comorbidity index and score for risk adjustment in hospital discharge abstracts using data from 6 countries. Am J Epidemiol.

[28] Virgilsen L, Moller H, Vedsted P (2019). Cancer diagnostic delays and travel distance to health services: a nationwide cohort study in Denmark. Cancer Epidemiol.

[29] Koo MM, Hamilton W, Walter FM, Rubin GP, Lyratzopoulos G (2018). Symptom signatures and diagnostic timeliness in cancer patients: a review of current evidence. Neoplasia.

[30] Lyratzopoulos G, Wardle J, Rubin G (2014). Rethinking diagnostic delay in cancer: how difficult is the diagnosis?. BMJ.

[31] Lyratzopoulos G, Abel GA, McPhail S, Neal RD, Rubin GP (2013). Measures of promptness of cancer diagnosis in primary care: secondary analysis of national audit data on patients with 18 common and rarer cancers. Br J Cancer.

[32] Zou G (2004). A modified poisson regression approach to prospective studies with binary data. Am J Epidemiol.

[33] Barros AJ, Hirakata VN (2003). Alternatives for logistic regression in cross-sectional studies: an empirical comparison of models that directly estimate the prevalence ratio. BMC Med Res Methodol.

[34] Ekholm O, Gundgaard J, Rasmussen NK, Hansen EH (2010). The effect of health, socio-economic position, and mode of data collection on non-response in health interview surveys. Scand J Public Health.

[35] Forbes LJ, Warburton F, Richards MA, Ramirez AJ (2014). Risk factors for delay in symptomatic presentation: a survey of cancer patients. Br J Cancer.

[36] Andersen RS, Vedsted P, Olesen F, Bro F, Sondergaard J (2009). Patient delay in cancer studies: a discussion of methods and measures. BMC Health Serv Res.

[37] Falborg AZ, Vedsted P, Menon U, Weller D, Neal RD, Reguilon I, Harrison S, Jensen H (2020). Agreement between questionnaires and registry data on routes to diagnosis and milestone dates of the cancer diagnostic pathway. Cancer Epidemiol.

[38] Holtedahl K, Vedsted P, Borgquist L, Donker GA, Buntinx F, Weller D, Braaten T, Hjertholm P, Mansson J, Strandberg EL (2017). Abdominal symptoms in general practice: Frequency, cancer suspicions raised, and actions taken by GPs in six European countries. Cohort study with prospective registration of cancer. Heliyon.

[39] Smith CF, Drew S, Ziebland S, Nicholson BD (2020). Understanding the role of GPs’ gut feelings in diagnosing cancer in primary care: a systematic review and meta-analysis of existing evidence. Br J Gen Pract.

[40] Elnegaard S, Pedersen AF, Sand Andersen R, Christensen RD, Jarbøl DE (2017). What triggers healthcare-seeking behaviour when experiencing a symptom? Results from a population-based survey. BJGP Open.

[41] Macleod U, Mitchell ED, Burgess C, Macdonald S, Ramirez AJ (2009). Risk factors for delayed presentation and referral of symptomatic cancer: evidence for common cancers. Br J Cancer.

[42] Pedersen AF, Hansen RP, Vedsted P (2013). Patient delay in colorectal cancer patients: associations with rectal bleeding and thoughts about cancer. PLoS One.

[43] Hamilton W, Round A, Sharp D, Peters TJ (2005). Clinical features of colorectal cancer before diagnosis: a population-based case-control study. Br J Cancer.

[44] Barrett J, Hamilton W (2008). Pathways to the diagnosis of lung cancer in the UK: a cohort study. BMC Fam Pract.

[45] Hamilton W (2009). Five misconceptions in cancer diagnosis. Br J Gen Pract.

[46] O'Sullivan ED, Schofield SJ (2018). Cognitive bias in clinical medicine. J R Coll Phys Edinb.

[47] Koo MM, von Wagner C, Abel GA, McPhail S, Rubin GP, Lyratzopoulos G (2017). Typical and atypical presenting symptoms of breast cancer and their associations with diagnostic intervals: evidence from a national audit of cancer diagnosis. Cancer Epidemiol.

[48] Ramirez AJ, Westcombe AM, Burgess CC, Sutton S, Littlejohns P, Richards MA (1999). Factors predicting delayed presentation of symptomatic breast cancer: a systematic review. Lancet.

[49] Danish Cancer Society. English title: Cancer patient's need and experiences with the health care system during cancer diagnostics and treatment. Copenhagen: The Danish Cancer Society; 2017.

[50] Evans J, Ziebland S, MacArtney JI, Bankhead CR, Rose PW, Nicholson BD (2018). GPs’ understanding and practice of safety netting for potential cancer presentations: a qualitative study in primary care. Br J Gen Pract.

[51] Danish Data protection Agency (2020). Danish data protection legislation.

[52] Intersoft Consulting (2021). General Data Protection Regulation.

[53] National Committee on Health Research Ethics (2018). Act on Research Ethics Review of Health Research Projects.

