# Supplementary material for: Alignment between the patient’s cancer worry and the GP’s cancer suspicion and the association with the interval between first symptom presentation and referral: a cross-sectional study in Denmark
Source: BMC Fam Pract. 2021 Jun 24;22:129. doi: 10.1186/s12875-021-01480-2 (PMC8228922; doi:10.1186/s12875-021-01480-2)
Supplement: Supplementary file 1 — Additional file 1 : Table 1: Categorisation of cancer types according to how difficult they are to diagnose clinically, based upon number of contacts in primary care before diagnosis in England [13, 28–30]. [file 12875_2021_1480_MOESM1_ESM.docx]

Additional file 1

Table 1: Categorisation of cancer types according to how difficult they are to diagnose clinically, based upon number of contacts in primary care before diagnosis in England [1-3]

| **Easy to diagnose** | | **Intermediate to diagnose** | | **Hard to diagnose** | |
| --- | --- | --- | --- | --- | --- |
| Rectal | (C19-20) | Oropharyngeal | (C10) | Stomach | (C16) |
| Melanoma* | (C43) | Oesophagus | (C15) | Pancreas | (C25) |
| Breast | (C50) | Colon | (C18) | Lung | (C34) |
| Vaginal | (C52) | Laryngeal | (C32) | Ovarian | (C56) |
| Cervical | (C53) | Mesothelioma | (C45-46) | Brain/CNS*** | (C70-72) |
| Endometrial | (C54-55) | Vulva | (C51) | Hodgkin’s Lymphoma | (C81) |
| Penile | (C60) | Prostate | (C61) | Multiple myeloma | (C90) |
| Testicular | (C62) | Renal | (C64) |  |  |
| Bladder | (C67) | NH Lymphoma** | (C82,83,85) |  |  |
| Thyroid | (C74-75) | Leukemia | (C91-C95) |  |  |

*Malignant melanoma

**Non-Hodgkin’s Lymphoma

***CNS: central nervous system
