# Supplementary material for: Alignment between the patient’s cancer worry and the GP’s cancer suspicion and the association with the interval between first symptom presentation and referral: a cross-sectional study in Denmark
Source: BMC Fam Pract. 2021 Jun 24;22:129. doi: 10.1186/s12875-021-01480-2 (PMC8228922; doi:10.1186/s12875-021-01480-2)
Supplement: Supplementary file 2 — Additional file 2 Prevalence rate ratio (PRR) of having long PCI according to the alignment between patient (PT) and general practitioner (GP) at the first clinical encounter and patient characteristics excluding gender-specific cancer types (n = 1639). [file 12875_2021_1480_MOESM2_ESM.docx]

***Additional file 2:*** *Prevalence rate ratio (PRR) of having long PCI according to the alignment between patient (PT) and general practitioner (GP) at the first clinical encounter and patient characteristics* ***excluding gender-specific cancer*** *types (n=1,639)*

|  | **PRR of having long PCI** | | |
| --- | --- | --- | --- |
|  | **Adjusted** | |  |
|  | **PRR** | **95% CI** |  |
| **Alignment between PT and GP** |  |  |  |
| 1.PT worried, GP suspicious | 1 |  |  |
| 2.PT *not* worried, GP suspicious | 1.16 | (0.95-1.41) |  |
| 3.PT worried, GP *not* suspicious | **3.35** | **(2.77-4.05)** |  |
| 4.PT *not* worried, GP *not* suspicious | **3.66** | **(3.03-4.44)** |  |
| **Sex** |  |  |  |
| Male | 1 |  |  |
| Female | 1.00 | (0.85-1.17) |  |
| **Age groups (years)** |  |  |  |
| 40-49 | 1 |  |  |
| >50-59 | 1.11 | (0.90-1.38) |  |
| >60-69 | 1.21 | (0.99-1.48) |  |
| >70-79 | 1.29 | (0.99-1.68) |  |
| >80 | **1.52** | **(1.22-1.87)** |  |
| **Year of diagnosis** |  |  |  |
| 2010 | 1 |  |  |
| 2016 | 0.86 | (0.78-0.95) |  |
| **Marital status** |  |  |  |
| Cohabiting/married | 1 |  |  |
| Not married | 1.04 | (0.93-1.15) |  |
| **Education** |  |  |  |
| Short | 1 |  |  |
| Medium | **0.85** | **(0.75-0.95)** |  |
| Long | **0.88** | **(0.78-0.99)** |  |
| **Diagnostic difficulty** |  |  |  |
| Easy | 1 |  |  |
| Intermediate | **1.21** | **(1.08-1.45)** |  |
| Hard | **1.35** | **(1.20-1.53)** |  |
| **Charlson’s Comordibity Index score** | | |  |
| None (0) | 1 |  |  |
| Low (1-2) | 0.98 | (0.83-1.15) |  |
| High (2 or more) | 1.15 | (0.93-1.43) |  |

**Adjusted for age, sex, year of diagnosis, marital status, education, diagnostic difficulty and CCI score*
