# Supplementary material for: Alignment between the patient’s cancer worry and the GP’s cancer suspicion and the association with the interval between first symptom presentation and referral: a cross-sectional study in Denmark
Source: BMC Fam Pract. 2021 Jun 24;22:129. doi: 10.1186/s12875-021-01480-2 (PMC8228922; doi:10.1186/s12875-021-01480-2)
Supplement: Supplementary file 3 — Additional file 3 Adjusted* prevalence rate ratio (PRR) of having long PCI according to the alignment between patient (PT) and general practitioner (GP) in the first clinical encounter and patient characteristics stratified on cancer type (n = 3333). [file 12875_2021_1480_MOESM3_ESM.docx]

**Additional file 3:** *Adjusted* prevalence rate ratio (PRR) of having long PCI according to the alignment between patient (PT) and general practitioner (GP) in the first clinical encounter and patient characteristics* ***stratified on cancer type (n=3,333)***

|  | **PRR of having long PCI** | | | | | | | | | | | |
| --- | --- | --- | --- | --- | --- | --- | --- | --- | --- | --- | --- | --- |
|  | **Colorectal (n=543)** | | **Lung (n=284)** | | **Melanoma (n=235)** | | **Breast (n=608)** | | **Prostate (n=491)** | | **Other (n=1172)** | |
|  | **PRR*** | **95% CI** | **PRR*** | **95% CI** | **PRR*** | **95% CI** | **PRR*** | **95% CI** |  |  |  |  |
|  | ****Long PCI: >16 days** | | **Long PCI: >37 days** | | **Long PCI: >8 days** | | **Long PCI: >0 days***** | | **Long PCI: >30 days** | | **Long PCI: > 20 days** | |
| **Alignment between PT and GP** |  |  |  |  |  |  |  |  |  |  |  |  |
| 1 .PT worried, GP suspicious | 1 |  | 1 |  | 1 |  | 1 |  | 1 |  | **1** |  |
| 2. PT *not* worried, GP suspicious | 1.12 | (0.82-1.54) | 0.54 | (0.29-1.00) | 0.95 | (0.41-2.21) | 1.51 | (0.52-4.38) | 0.93 | (0.57-1.53) | 1.13 | (0.70-1.83) |
| 3. PT worried, GP *not* suspicious | **4.75** | **(3.58-6.31)** | **2.93** | **(1.81-4.75)** | **4.06** | **(2.57-6.43)** | **6.64** | **(4.30-10.27)** | **2.41** | **(1.59-3.66)** | **4.17** | **(2.90-6.00)** |
| 4. PT *not* worried, GP *not* suspicious | **4.99** | **(3.60-6.93)** | **3.88** | **(2.45-6.12)** | **4.14** | **(2.44-6.74)** | **5.94** | **(3.47-10.15)** | **2.06** | **(1.45-2.91)** | **4.50** | **(3.30-6.12)** |
| **Sex** |  |  |  |  |  |  |  |  |  |  |  |  |
| Male | 1 |  | 1 |  | 1 |  |  |  |  |  |  |  |
| Female | 1.08 | (0.78-1.49) | 0.93 | (0.68-1.29) | 0.94 | (0.61-1.44) | NA |  | NA |  | 0.89 | (0.76-1.05) |
| **Age groups (year)** |  |  |  |  |  |  |  |  |  |  |  |  |
| 40-49 | 1 |  | 1 |  | 1 |  | 1 |  | 1 |  | 1 |  |
| >50-59 | 0.99 | (0.64-1.53) | 0.79 | (0.19-3.23) | 1.07 | (0.68-1.69) | 0.82 | (0.47-1.45) | 0.38 | (0.25-0.57) | **1.38** | **(1.05-1.82)** |
| >60-69 | 1.33 | (0.91-1.95) | 0.98 | (0.31-3.12) | 0.81 | (0.51-1.29) | 1.06 | (0.64-1.76) | 0.26 | (0.18-0.39) | **1.56** | **(1.22-1.99)** |
| >70-79 | 1.09 | (0.63-1.89) | 1.44 | (0.46-4.55) | **0.48** | **(0.27-0.86)** | 0.82 | (0.38-1.75) | 0.38 | (0.28-0.52) | **1.76** | **(1.39-2.24)** |
| >80 | **1.67** | **(1.10-2.54)** | 1.19 | (0.43-3.34) | 1.60 | (0.63-4.04) | 0.74 | (0.34-1.61) | 0.44 | (0.27-0.73) | **1.66** | **(1.04-2.64)** |
| **Year of diagnosis** |  |  |  |  |  |  |  |  |  |  |  |  |
| 2010 | 1 |  | 1 |  | 1 |  | 1 |  | 1 |  | 1 |  |
| 2016 | **0.68** | **(0.53-0.87)** | 1.25 | (0.92-1.68) | 0.84 | (0.54-1.30) | 1.16 | (0.88-1.54) | 0.97 | (0.77-1.23) | 0.89 | (0.77-1.02) |
| **Marital status** |  |  |  |  |  |  |  |  |  |  |  |  |
| Married | 1 |  | 1 |  | 1 |  | 1 |  | 1 |  | 1 |  |
| Not married | 1.17 | (0.96-1.42) | 1.24 | (0.89-1.74) | 0.96 | (0.71-1.30) | 1.31 | (0.94-1.83) | **0.73** | **(0.56-0.96)** | 1.00 | (0.77-1.31) |
| **Education** |  |  |  |  |  |  |  |  |  |  |  |  |
| Short | 1 |  | 1 |  | 1 |  | 1 |  | 1 |  | 1 |  |
| Medium | 1.07 | (0.79-1.43) | **0.75** | **(0.57-0.97)** | 0.69 | (0.47-1.00) | 0.79 | (0.51-1.22) | **1.37** | **(1.03-1.82)** | 0.85 | (0.73-0.99) |
| Long | 0.83 | (0.53-1.31) | **0.41** | **(0.23-0.70)** | **0.60** | **(0.41-0.98)** | 1.08 | (0.73-1.61) | **1.39** | **(1.02-1.90)** | 1.00 | (0.80-1.27) |
| **Charlson’s Comordibity Index score** | | |  |  |  |  |  |  |  |  |  |  |
| None (0) | 1 |  | 1 |  |  |  | 1 |  | 1 |  | 1 |  |
| Low (1-2) | 1.06 | (0.87-1.29) | **1.63** | **(1.23-2.16)** | 0.85 | (0.49-1.47) | **1.94** | **(1.39-2.73)** | 0.98 | (0.73-1.30) | 0.93 | (0.76-1.14) |
| High (2 or more) | 1.74 | (1.15-2.63) | 1.10 | (0.88-1.38) | 1.17 | (0.17-7.91) | **1.98** | **(1.11-3.53)** | 0.99 | (0.53-1.83) | 0.92 | (0.50-1.70) |

**Adjusted for age, sex, year of diagnosis, marital status, education and CCI score*

NA: not applicable

**Long PCI was defined as an interval longer than the 75th percentile for each cancer type using a pseudo variable [4] for PCI as some of the percentiles had fewer than 5 observations violating rules of reporting according to Statistics Denmark.

*** Long PCI for breast cancer patients was rounded to 1 day.

**References**

1. Lyratzopoulos G, Wardle J, Rubin G: **Rethinking diagnostic delay in cancer: how difficult is the diagnosis?** *BMJ (Clinical research ed)* 2014, **349**:g7400.

2. Lyratzopoulos G, Neal RD, Barbiere JM, Rubin GP, Abel GA: **Variation in number of general practitioner consultations before hospital referral for cancer: findings from the 2010 National Cancer Patient Experience Survey in England**. *The LancetOncology* 2012, **13**(4):353-365.

3. Koo MM, Hamilton W, Walter FM, Rubin GP, Lyratzopoulos G: **Symptom Signatures and Diagnostic Timeliness in Cancer Patients: A Review of Current Evidence**. *Neoplasia* 2018, **20**(2):165-174.

4. **Pseudo percentiles** [<http://w.bruunisejs.dk/StataHacks/Datamanagement/pseudo_percentiles/pseudo_percentiles/>]
